# Plasmonic Nanopillars—A Brief Investigation of Fabrication Techniques and Biological Applications

**DOI:** 10.3390/bios13050534

**Published:** 2023-05-10

**Authors:** Heesang Ahn, Soojung Kim, Sung Suk Oh, Mihee Park, Seungchul Kim, Jong-ryul Choi, Kyujung Kim

**Affiliations:** 1 Department of Cogno-Mechatronics Engineering, Pusan National University, Busan 46241, Republic of Korea; ahn3890@pusan.ac.kr (H.A.); kimsoo9640@pusan.ac.kr (S.K.); s.kim@pusan.ac.kr (S.K.); 2 Medical Device Development Center, Daegu-Gyeongbuk Medical Innovation Foundation (K-MEDI hub), Daegu 41061, Republic of Korea; ssoh@kmedihub.re.kr; 3 Educational Research Center for the Personalized Healthcare based on Cogno-Mechatronics, Pusan National University, Busan 46241, Republic of Korea; mhpark@pusan.ac.kr; 4 The Department of Optics and Mechatronics Engineering, Pusan National University, Busan 46241, Republic of Korea

**Keywords:** plasmonic nanopillars, localized surface plasmon resonance, optical sensing, enhanced Raman spectroscopy, high-resolution optical imaging

## Abstract

Nanopillars (NPs) are submicron-sized pillars composed of dielectrics, semiconductors, or metals. They have been employed to develop advanced optical components such as solar cells, light-emitting diodes, and biophotonic devices. To integrate localized surface plasmon resonance (LSPR) with NPs, plasmonic NPs consisting of dielectric nanoscale pillars with metal capping have been developed and used for plasmonic optical sensing and imaging applications. In this study, we studied plasmonic NPs in terms of their fabrication techniques and applications in biophotonics. We briefly described three methods for fabricating NPs, namely etching, nanoimprinting, and growing NPs on a substrate. Furthermore, we explored the role of metal capping in plasmonic enhancement. Then, we presented the biophotonic applications of high-sensitivity LSPR sensors, enhanced Raman spectroscopy, and high-resolution plasmonic optical imaging. After exploring plasmonic NPs, we determined that they had sufficient potential for advanced biophotonic instruments and biomedical applications.

## 1. Introduction

Surface plasmon resonance (SPR) is a phenomenon wherein a surface plasmon is excited by a light incident on a metal-dielectric surface [1,2,3,4,5,6,7]. To concentrate the electromagnetic fields generated under SPR and enhance performance, localized surface plasmon resonance (LSPR), which is defined as SPR that occurs in nanoscale metallic structures, has been investigated [8,9,10,11,12,13,14,15]. LSPR has been used for developing various optical components, advanced display systems, and biomedical devices. To optimize LSPR, research groups have designed and developed several nanostructures and nanoparticles according to their fields of application.

A nanopillar is defined as an array of submicron-sized pillars composed of dielectrics, semiconductors, or metals. They have been used to develop advanced optical components such as flexible perovskite solar cells [16,17,18,19,20,21] and waveguide-coupled light-emitting diodes (LEDs) [22,23,24,25,26]. In cell-based assays, nanopillar (NP)-fabricated cell culture substrates are used to acquire valuable biological information from the membranes of cultured cells [27,28,29,30,31,32]. Xie et al. [29] conducted a representative study wherein they developed a platinum NP array for application as an electrode to record the intracellular action potentials of cultured cells on the NPs. Hanson et al. [30] utilized a transparent dielectric NP array as a nanoscale optical light guide to obtain the nuclear mechanics in some cultured cells by optical fluorescence imaging. Additionally, when these NPs were capped with metal, well-localized SPR could be generated; plasmonic NPs have been employed for advanced optical applications based on optimized LSPR. In this study, plasmonic NPs have been defined as nanophotonic structures consisting of dielectric pillars with metal capping for plasmonic enhancements. Meanwhile, several research groups have referred to plasmonic NPs as nano-mushrooms because the metallic cap and dielectric pillar in plasmonic NPs resemble the cap and stem of a mushroom, respectively.

In this study, we describe the fabrication techniques for plasmonic NPs and their applications in relation to enhancements in the performance of optical sensing and imaging. We explained three fabrication methods to produce an NP array: etching based on semiconductor fabrication processes, nanoimprinting, and growth of NPs on a substrate. Additionally, the role of metal capping in providing plasmonic enhancement was briefly described. Then, we described the applications of plasmonic NPs, including highly sensitive optical sensors using plasmonic NPs, plasmonic NP-enhanced Raman spectroscopy, and high-resolution optical imaging based on plasmonic enhancement by NPs. Following this, we presented several ways to improve the fabrication and application of plasmonic NPs. We believe that this study will be helpful for future studies and applications regarding plasmonic NPs.

## 2. Fundamental Principle of Plasmonic NPs

Plasmonic nanopillars to improve the sensitivity of optical sensors or to obtain high-resolution optical images are based on surface plasmon resonance (SPR) and localized surface plasmon resonance (LSPR). Surface plasmons (SPs) are collective vibrations of free electrons in a region confined to a boundary between metal and dielectric [33,34,35,36,37]. These vibrating electrons interact with light (photons) to create a state called SPR. Electronic SPs follow the dispersion relation as follows:

(1) $$k\left(\omega \right)=\frac{\omega }{c}\sqrt{\frac{\varepsilon_{1}\varepsilon_{2}}{\varepsilon_{1}+\varepsilon_{2}}}$$

where *k(w)* is the wave vector, *w* is the angular frequency, and *c* is the speed of light. ε_1_ and ε_2_ indicate the relative permittivities of dielectric and metal, respectively.

Subwavelength plasmonic nanopillars correspond to metallic nanostructured surfaces with a size smaller than the wavelength of light, thus generating LSPR, one of the SPRs. LSPR is an SPR that occurs between a metal nanostructure with a size smaller than the wavelength of light and a dielectric, which induces locally strong electromagnetic field concentration and significantly improves optical properties such as sensitivity in plasmonic sensors [38,39,40].

On the other hand, LSPR optical sensors using nanostructures that are directly in contact with a substrate have the problem that a part of the electromagnetic fields, which should be concentrated on the nanostructure, is transmitted to the substrate [41]. Plasmonic nanopillars with a dielectric pillar make a constant interval between the substrate and the nanostructure in which the electromagnetic field should be concentrated [42]. It provides a uniform dielectric environment so that electromagnetic fields can be more concentrated on the desired nanostructure. It contributes to improving optical characteristics such as sensitivity.

Plasmonic nanopillars, which are used for high-resolution optical imaging of biological molecules in cells, simultaneously utilize the characteristics of localized light delivery and plasmonic enhancement of fluorescence excitation by both subwavelength optical waveguides and LSPR.

## 3. Fabrication Techniques for Plasmonic NPs

The methods involved in the fabrication of plasmonic NP arrays can be divided into two processes, namely NP fabrication and metallic deposition, wherein the tip of NPs is capped with metal. Regarding NP fabrication, an etching-based fabrication process, nanoimprinting lithography, and a growth method can be used. In this section, the techniques used to fabricate plasmonic NPs and metal deposition, which are used in plasmonic enhancement, were explained.

### 3.1. Etching Based on Semiconductor Fabrication Processes

**Figure 1:**
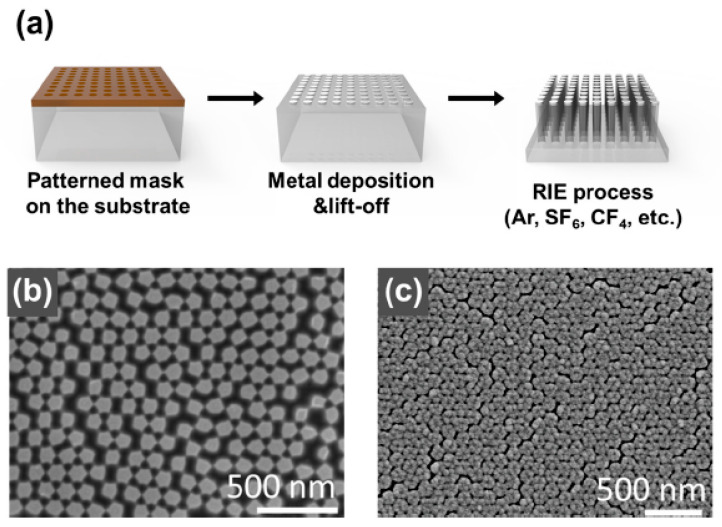
(**a**) Overview of reactive-ion etching (RIE) processes for fabrication of a plasmonic nanopillar (NP) array. (**b**) Scanning electron microscope (SEM) images of Si nanofiller arrays manufactured by template-assisted ion etching techniques. (**c**) SEM images of Ag nano-mushroom arrays after depositing Ag layers on Si NPs. Reprinted with permission from Ref. [47]. Copyright 2022, Springer Nature.

In numerous studies, the combination of dry etching methods with different masking techniques has been utilized for the production of nanoparticles, particularly in materials such as Si, SiN, SiO_2_, and sapphire substrates. To fabricate nanoparticles, various nano-sized masks are created on a substrate using lithography techniques such as electron-beam lithography [43,44,45] and microsphere lithography [46,47,48,49]. Following this, dry etching using gases and reactive-ion etching (RIE) is carried out. This process offers several advantages, including high resolution, reproducibility, high finishing accuracy, and the ability to process hard materials. However, the fabricated structure’s aspect ratio is limited, and the output of the process depends on the gas used during the process. Kim et al. [43] reported the formation of NPs in thin quartz films. In their experiments, they used electron-beam lithography and RIE (Figure 1). Initially, poly(methyl methacrylate) (PMMA) (Allresist GmbH, AR-P 672.045) for the electron-beam lithography resists and a conducting polymer (Allresist GmbH, AR-PC 5090.02) were spin-coated on the cleaned quartz substrate at 180 and 95 °C, respectively, to remove the residual solvent. The polymer was used as a charge dissipation layer when electron-beam lithography was applied to a quartz substrate, which is an insulator. The annealed substrate was patterned by electron-beam lithography, the conducting polymer was removed in deionized water, and PMMA was developed with isopropyl alcohol and methyl isobutyl ketone in a ratio of 3:1. The etching mask was formed by electron-beam evaporation of 50 nm of Cr, followed by lift-off with acetone and sonication. The quartz with the Cr mask was subjected to RIE with 7 sccm of Ar and 5 sccm of CF_4_ for 3 h at a radio-frequency power of 50 W. They obtained an NP array with a diameter of 200 nm, a height of 700 nm, and a period of 1 μm. Other researchers [44] used gold masks made by electron-beam lithography on a quartz substrate and fabricated NPs through RIE with O_2_ and CHF_3_ gases.

Xu et al. [46] reported a nanopillar fabrication process using multi-patterning nanosphere lithography that combined nanosphere lithography, metallic masks, and RIE. In the fabrication process, the polystyrene nanospheres with diameters ranging from 0.4 to 2 μm were arranged in a monolayer on a substrate using a nanosphere dispensing system. The size of the nanospheres was controlled by etching them with O_2_ plasma. Then, the substrate was etched through deep RIE using C_4_F_8_ and SF_6_ gases to form hexagonally arranged nanopillar arrays. Furthermore, the size of the remaining nanospheres was adjusted using O_2_ plasma treatment, and a proposed nanotube fabrication process involved an additional etching process after depositing a metal mask and removing the nanospheres. He et al. [47] used a single layer of polystyrene as an etching mask and reported the production of nanopillars by RIE using SF_6_ and O_2_.

### 3.2. Nanoimprinting

**Figure 2:**
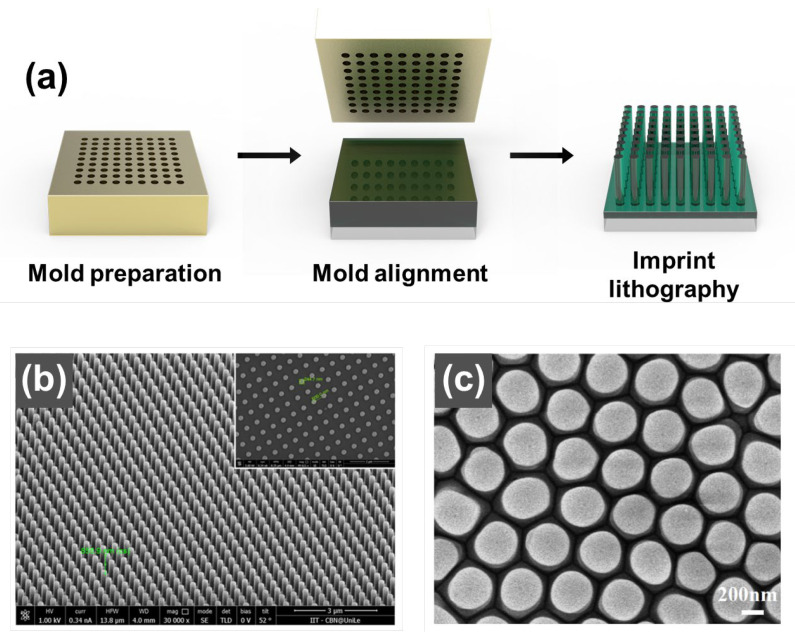
(**a**) Overview of the nanoimprint lithography process for fabrication of a plasmonic nanopillar (NP) array. (**b**) SEM image of sub-micron PMMA pillars; (inset) a top-view image of the PMMA plasmonic nano-pillar array. Reprinted with permission from Ref. [51]. Copyright 2019, MDPI. (**c**) SEM image of arrays of polycarbonate pillars. Reproduced with permission from Ref. [53]. Copyright 2021, Elsevier B.V.

Nanoimprinting is a low-cost, high-resolution, and repeatable method for nanofabrication that involves transferring mold patterns onto resist films. In this experimental method, a patterned mold is pressed onto a thermoset polymer or UV-cured polymer-coated substrate while heat or ultraviolet (UV) rays are irradiated, resulting in pattern transfer (see Figure 2). The nanoimprinting process provides high throughput, accurate and repeatable replication, and low cost. However, the range of materials that can be used to fabricate nanopillars is limited to UV-curable resins, polymers, and thermally fluid materials [50,51,52,53].

Xu et al. [50] reported the fabrication of NP patterns using nanoimprinting with nickel molds in flexible, lightweight polymers. In another example, an NP was fabricated using a PMMA or polydimethylsiloxane (PDMS) stamp to make an NP with a diameter of 100 nm [51,52].

Che et al. [53] reported the fabrication of ordered polymer nanopillar arrays using a combination of AAO templates and nanoimprinting. In the first step, polycarbonate particles on a glass substrate were heated to 300 °C to form a film with fluidity. Then, a single-pass AAO template was placed on top of the polycarbonate film at 300 °C for nanopillar formation, followed by continuous pressure. After cooling to room temperature, the AAO template was removed using a mixture of 3.4 g CuCl_2_, 75 mL HCl, and 100 mL H_2_O. The polycarbonate nanopillars were formed in uniform dimensions, with a diameter of 380 nm and a spacing of 70 nm.

### 3.3. Growth of NPs on a Substrate

**Figure 3:**
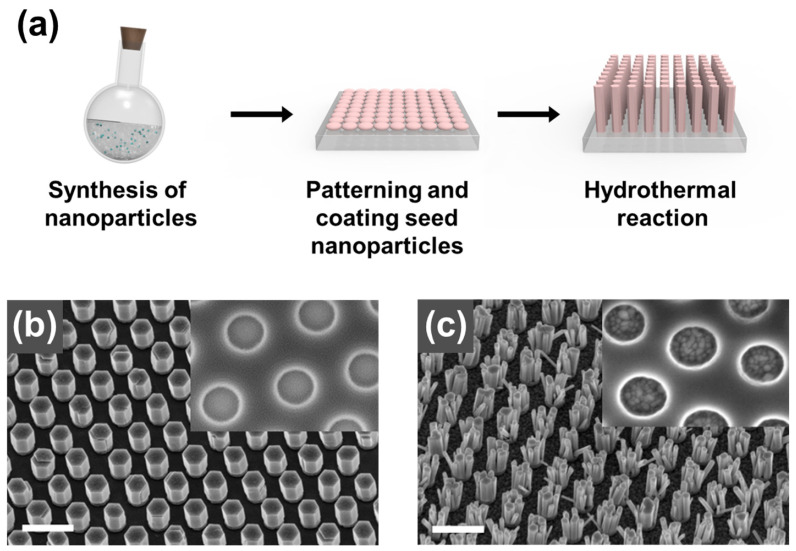
(**a**) Overview of the growth process for the fabrication of a plasmonic nanopillar (NP) array. ZnO nanowires were grown on (**b**) single-crystalline ZnO substrates and (**c**) poly-crystalline ZnO thin films. Reprinted with permission from Ref. [55]. Copyright 2012, Elsevier B.V.

The fabrication of metal oxide pillar structures utilizing the growth of nanoparticles (NPs) on a substrate has been a commonly employed method [54,55,56]. While the process offers low-cost fabrication and high aspect ratios, it suffers from low reproducibility and uniformity in the substrate unit. Hydrothermal synthesis is a representative method used to synthesize crystals in an aqueous solution, with the synthesized crystals being influenced by factors such as temperature, the concentration of the solution, growth time, and crystallographic direction of the substrate. This method offers advantages such as simplicity, low temperature, and cost-effectiveness. Cui et al. [54] reported the formation of an NP of ZnO in PET and Si wafers (Figure 3). In the synthesis process, ZnO nanoparticle seeds were first coated onto a Si substrate. A KOH solution was added to the zinc acetate dihydrate solution at 60 °C. Then, the mixture was stirred for 3 h to produce a ZnO seed. Only a specific area of the substrate was coated with the prepared seed via screen printing. Then, the printed substrate was inverted in an aqueous solution at 70–100 °C for 3.5 h. The obtained patterned ZnO NPs were then washed several times with water and dried, producing a hexagonal ZnO NP with high-quality crystals.

### 3.4. Metal Capping for Plasmonic Enhancement

Non-capped dielectric NPs have high-aspect-ratio waveguide characteristics. However, they have limitations as probes because of their diffraction-limited imaging resolution. A metallic plasmonic cap can improve their resolution and sensitivity by localizing light through LSPR, thereby overcoming these limitations. The NPs with plasmonic capping used for LSPR sensing are more sensitive to changes in the refractive index around the metallic structure than SPR without nanostructures are, and they can observe chemical or biological changes in the surrounding reflective index more sensitively. Thermal evaporation and electron-beam evaporation are generally employed to evenly deposit metallic caps with nanoscale thicknesses. Li et al. [57] applied thermal evaporation to the fabrication of metallic caps consisting of nickel with a thickness of 5 nm and gold with a thickness of 110 nm. Xu et al. [50] deposited Ag on NPs using electron-beam evaporation. Sputtering by energetic plasma particles has also been used for metal capping on NPs. As a representative case, He et al. [47] fabricated plasmonic NPs in a microfluidic plasmonic sensor using both etching and large-area sputtering.

## 4. Applications of Plasmonic NPs

### 4.1. Highly Sensitive LSPR Sensors Using Plasmonic NPs

Generally, LSPR generated by well-designed nanostructures or nanoparticles has better sensitivity to changes in the surrounding refractive indices than SPR under certain conditions of incident-light wavelengths. Based on this characteristic, LSPR-based highly sensitive plasmonic sensors have the following merits over other sensors: high sensitivity to detect very low concentrations of the specimen; fast sensing response speed to changes in concentration or property of samples; non-destructive analysis; and miniaturization of the entire sensing system using compact light sources and photodetectors. On account of these advantages, LSPR has been actively applied in the development of highly sensitive chemical sensors and biosensors [58,59,60,61,62,63,64,65]. Several research groups have used plasmonic NPs as nanostructures to generate enhanced LSPR in highly sensitive chemical sensors and biosensors.

**Figure 4:**
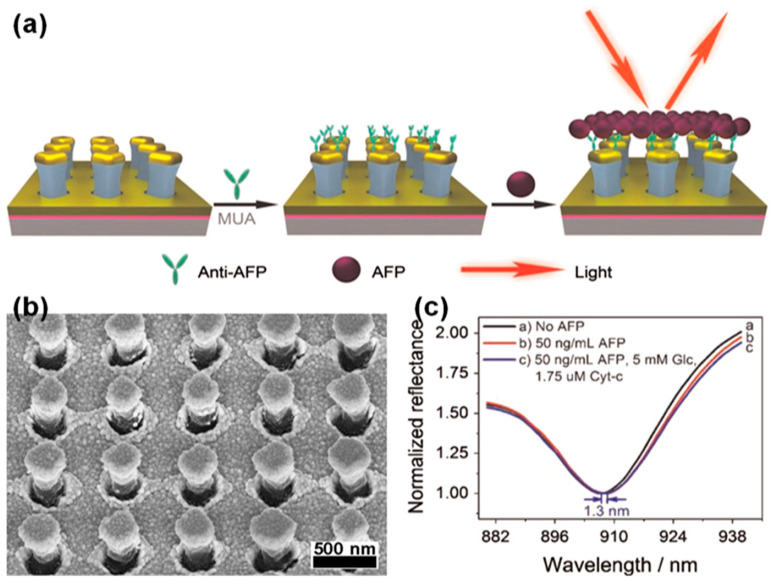
Development of a LSPR-based plasmonic biosensor using a plasmonic NP array with gold capping for the highly sensitive detection of alpha-fetoprotein (AFP). (**a**) Schematic diagram of one-step AFP detection using an LSPR spectroscopic sensor with plasmonic NPs. (**b**) SEM image of the fabricated plasmonic NP array. (**c**) Reflectance spectra were measured for the following: (1) no AFP, (2) 50 ng/mL of AFP, and (3) a mixture of 50 ng/mL of AFP, 5 mm of glucose, and 1.75 μM of cyt c for 30 min. Reprinted with permission from Ref. [57]. Copyright 2015, Elsevier B.V.

Li et al. [57] employed a plasmonic NP array with gold capping to develop an LSPR-based plasmonic biosensor for the highly sensitive detection of alpha-fetoprotein (AFP), which is a biomarker of liver cancer, as shown in Figure 4. Researchers have produced a substrate of plasmonic NPs capped with 5 nm of nickel (as an adhesive layer) and 110 nm of gold on dielectric NPs made of positive photoresist, as shown in Figure 4a,b. Anti-AFP functionalization was performed to ensure that the AFP adhered well. Researchers have tested the performance of optical biosensors using plasmonic NPs with solutions having different refractive indices; they obtained a refractive index sensitivity of 465 nm/RIU. The sample measurement for various AFP concentrations revealed that the developed AFP sensor enhanced by the plasmonic NPs was sensitive to an AFP concentration of 0.008 nm/(ng/mL) in the range of 10 to 200 ng/mL; the limit of detection (LOD) was 24 ng/mL. The results of this study indicated that plasmonic NPs improved both refractive index sensitivity and penetration depth, enabling AFP molecules to be measured more effectively. Additionally, researchers [66] have applied plasmonic NPs designed for highly sensitive AFP biosensors to transmissive and multi-chamber biosensors. To fabricate large-area transmissive biosensors, a transparent optical adhesive (NOA63) was applied to PDMS nanoscale stamps. Subsequently, a transparent dielectric NP array was created by hardening with UV exposure. Nickel (5 nm) and gold (110 nm) were capped on the NP arrays to develop transmissive LSPR biosensors. Researchers measured the transmission spectra and variations in the peak wavelength of plasmonic NP-integrated transmissive biosensors using solutions with different refractive indices, and the measured refractive index sensitivity was 565 nm/RIU. To confirm the feasibility of biosensors in simultaneously measuring multiple biomarkers, a plasmonic NP-integrated LSPR biosensor with four chambers was configured. Glycerol saline solution, alcohol-containing disinfectant, medical glucose injection solution, and cholesterol in human serum were applied to the multi-chamber LSPR sensor to measure the differences in the transmission spectra; the LODs for the glucose and cholesterol were 67.9 and 92.5 mg/dL, respectively. The results showed that plasmonic NP-integrated LSPR biosensors had the potential to be applied to medical sensors and highly sensitive Raman spectroscopic measurements.

Jiao et al. [67] developed a plasmonic antibody-antigen sensor using plasmonic NP array metasurfaces composed of aluminum and a flexible polymer. Regarding the plasmonic NPs used in this research, flexible polymer NPs were fabricated using nanoimprinting with a nickel basement and were then covered with aluminum. Researchers have compared the refractive index sensitivities of plasmonic NPs with gold and aluminum capping. The sensors with the gold-covered and aluminum-covered plasmonic NP substrates had refractive index sensitivities of 424 and 553 nm/RIU, respectively. Based on these results, we developed an LSPR-based optical biosensor with an aluminum-capped plasmonic NP array. The plasmonic biosensor had an LOD of less than 1 pg/mL for bovine serum albumin (BSA)/anti-BSA interactions. In a comparative study on gold and aluminum capping, the biosensors with gold- and aluminum-capped plasmonic NPs had maximum wavelength shifts of 2.70 ± 0.23 and 6.43 ± 0.53 nm, respectively.

**Figure 5:**
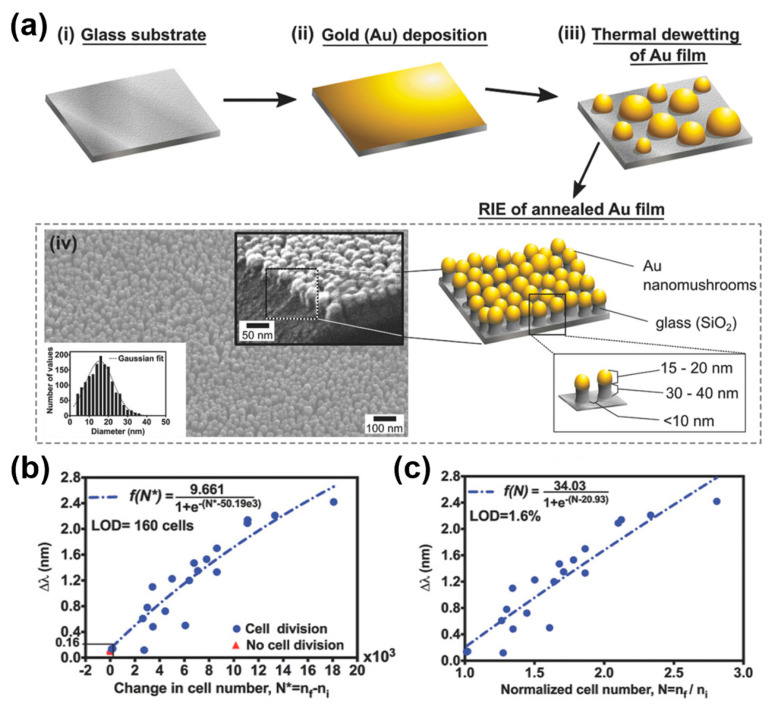
Transmissive optical spectroscopic measurements enhanced by LSPR of a plasmonic NP array to monitor cell proliferation. (**a**) Schematic of fabrication processes for the plasmonic NP array for highly sensitive and label-free cell proliferation monitoring. (**b**) LSPR responses (redshifts) according to changes in the numbers of cells during a cell proliferation period of 24–168 h. (**c**) Redshifts by the normalized number of cells cultured on the plasmonic NP array-fabricated substrate. Reprinted with permission from Ref. [68]. Copyright 2018, Wiley-VCH Verlag GmbH & Co. KGaA, Weinheim.

A study was also conducted wherein plasmonic NPs were utilized for the label-free analysis of the characteristics of cultured cells. Bhalla et al. [68] employed transmissive optical spectroscopic measurements enhanced by the LSPR of a plasmonic NP array to monitor cell proliferation processes, as shown in Figure 5. Researchers fabricated randomly distributed nanoislands with diameters of 15–20 nm by the thermal dewetting of a gold film substrate at 560 °C for 3 h and produced a plasmonic NP array consisting of dielectric pillars with heights of 30–40 nm and gold caps of 15–20 nm by RIE, as shown in Figure 5a. In the fabricated plasmonic NP array, the positions of each NP were irregular but arranged at close intervals of 10–20 nm. Tests on samples with different refractive indices indicated that the plasmonic NP array-based sensor with the transmissive optical spectroscopic measurement system developed by the researchers had a refractive index sensitivity of 83.15 ± 0.8998 nm/RIU. The optical transmission (absorption) spectra of cells cultured on a plasmonic NP-integrated substrate were measured over seven days (168 h), and the changes in peak wavelength caused by the proliferation processes confirmed by brightfield images showed the possibility of a highly sensitive cell-status monitor-sensor.

Additionally, several studies have been performed wherein improvements in the performance of plasmonic NP-enhanced LSPR sensors by theoretical simulation and artificial intelligence-based optimization have been discussed. Bhalla et al. [69] studied the LSPR phenomenon of randomly distributed plasmonic NPs and the differences in enhancements in LSPR by varying the specifications of NPs using unsupervised machine learning. Regarding the design of plasmonic NP array-based LSPR sensors, it is expected that predictive results using an artificial intelligence-based algorithm can be useful. Agrawal et al. [70] studied localized electromagnetic field distributions and sensing characteristic improvements by applying a plasmonic NP array as a substrate for near-infrared SPR refractive index sensors through rigorous coupled wave analysis, one of the simulation methods of plasmonic fields. Researchers calculated the refractive index sensitivity and figure-of-merit by setting the height of the dielectric pillars and the distance between the golden caps as variables. The simulation results indicated that an optimized plasmonic NP array and an integrated near-infrared refractive index sensor with a wavelength band of 1,000 nm had a sensitivity of 9,920 nm/RIU and a figure-of-merit of 58.2 RIU^−1^. Researchers have also suggested fabrication processes for optimized plasmonic NP arrays using electron-beam lithography and isotropic etching.

### 4.2. Plasmonic NP-Enhanced Raman Spectroscopy

Raman spectroscopy is a spectroscopic modality used to measure the vibrational mode of molecules [71,72,73,74,75] and has been applied as a nondestructive molecular measurement method to determine the presence of materials with specific molecular structures. Surface-enhanced Raman spectroscopy (SERS) allows Raman signals to be enhanced by nanoplasmonic light confinements [76,77,78,79,80,81]. Generally, metallic nanoparticles, silica-coated particles, periodic nanoapertures, and randomly distributed nanoislands are widely used in SERS [52,58,82,83,84,85]. Several studies on the application of plasmonic NPs for SERS-based high-sensitivity sensors have also been actively performed. 

**Figure 6:**
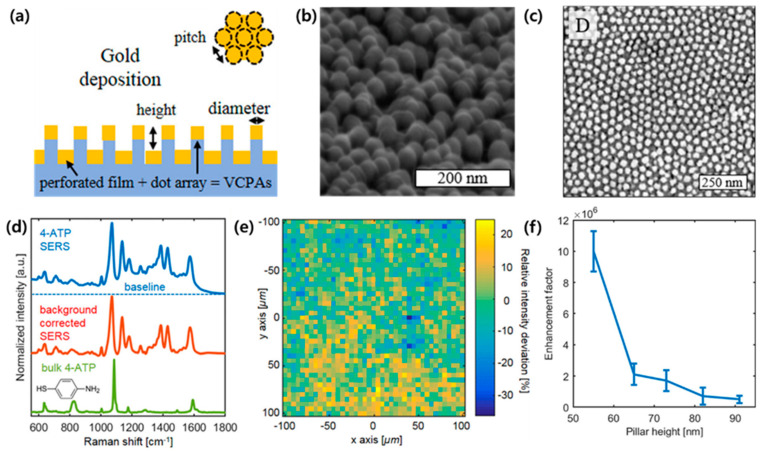
(**a**) A schematic of gold-covered nanopillars with small height-to-diameter aspect ratios for high-sensitive surface-enhanced Raman spectroscopy (SERS). (**b**,**c**) Scanning electron microscopy (SEM) images of the fabricated gold-covered nanopillars were acquired (b) at a 70° angle and (**c**) vertically. (**d**) Normalized SERS and conventional Raman spectra in a measurement of four-ATP. (**e**) A 200 μm × 200 μm Raman intensity deviation map at 1075 cm^−1^. The average relative standard deviation of Raman intensity was 8%. (**f**) A relationship between SERS enhancement factors and the height of plasmonic nanopillars. Reprinted with permission from Ref. [86]. Copyright 2020, American Society of Chemistry (ACS).

Akinoglu et al. [86] developed gold-covered nanopillars with small height-to-diameter aspect ratios as a SERS substrate (Figure 6a). The plasmonic nanopillars induced two types of stronger couplings: (i) lateral coupling between the tips of the nanopillars or (ii) vertical coupling of gold capping and deposited gold nanoparticles on the side walls of the nanopillars. The interaction presented an increase in SERS scattering efficiency. As shown in Figure 6b, the structure consists of a gold film deposited on the bottom of the nanopillars and a gold capping array on the top of the nanopillars. In this work, the gap between the gold film and gold capping was referred to as a plasmonic slab. The SERS enhancement of the nanopillars is determined by the geometric properties of the plasmonic slab. The height of the nanopillars can be modulated through etching process parameters. A small period compared to the diameter can increase the pillar density and cause lateral coupling between the vertices of the pillars, resulting in increased hotspot formation for SERS. In Figure 6c, the nanopillars have an average diameter of d = 33.7 nm ± 7.1 nm with a small standard deviation, reducing the inhomogeneous broadening of the plasmon resonance that can lower the SERS intensity. Figure 6d presents the normalized SERS spectrum and conventional Raman signal of bulk four-ATP that was selected as a probe, with the strongest peak occurring at −1075 cm^−1^. To demonstrate the reliability of the SERS signal measured in the plasmonic nanopillar system, the background-corrected SERS intensity was measured, and the spectrum showed an average relative standard deviation of 8% (Figure 6e). The SERS enhancement factor with various nanopillar heights was investigated (Figure 6f). Average values assume that the gold surface of the nanopillars is completely covered with four-ATP molecules and that all characters contribute to the Raman signal. The higher and smaller the slab spacing, the greater the SERS enhancement, which significantly increases with shorter heights of the nanopillars and smaller gaps of the plasmonic slab. The strongest enhancement factor is calculated as 1 × 10^7^ for nanopillars with 55 nm of height. The size of the plasmonic gaps applied in this work is large enough to functionalize the gold surface and has enough space to enable access to larger macromolecules such as proteins. 

Xu et al. [50] investigated silver-coated plasmonic NP substrates over a large area for SERS polymer sensors. Plasmonic NPs were fabricated on a four-inch wafer; specifically, they were obtained using a nanohole array substrate as a nanoimprinting mask to fabricate dielectric NPs, and silver was capped on them. Researchers have developed colorimetric refractive index sensors using changes in the reflection characteristics in the visible region. Additionally, a sensor of a monolayer of trans-1,2-bis(4-pyridyl)ethylene with Raman signals improved over 10 times was developed by applying the substrate, wherein plasmonic NPs were integrated into a commercial SERS measurement system. Through subsequent research, these researchers [87] developed a novel plasmonic NP array-integrated substrate that could further improve the sensitivity of Raman spectroscopy for antibody–antigen reactions. The plasmonic NP consisted of a dielectric NP, a spherical silver cap, and randomly distributed silver nanoislands on the side of the NP. A handheld-type SERS measurement system was investigated based on the improvement in sensitivity achieved using this plasmonic NP array. In a comparative study on Raman-peak measurements of 1 ppm of methamphetamine, a higher sensitivity was observed using plasmonic NPs surrounding the silver nanoislands. A study wherein gold was used instead of silver in plasmonic NPs indicated a higher improvement in sensitivity than when the gold standard for SERS, namely the Klarite substrate, was used; this improvement was achieved by optimizing the height of dielectric NPs [88].

**Figure 7:**
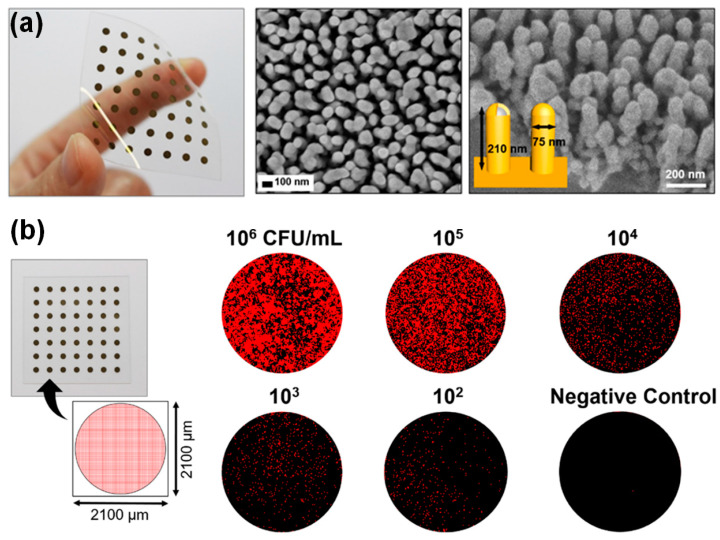
Investigation of a flexible and skin-attachable SERS sensor that can detect bacterial pathogens using both plasmonic NPs and gold nanoparticle tags. (**a**) Large-area plasmonic silver/gold-coated NP array on a flexible polymer film and SEM images of the plasmonic NPs. (**b**) Raman images of Salmonella in the range of 0 to 10^6^ CFU/mL. Reprinted with permission from Ref. [89]. Copyright 2018, American Chemical Society (ACS).

Plasmonic NP arrays fabricated on a polyethylene terephthalate (PET) film can be flexibly used as highly sensitive SERS biosensors. Ko et al. [89] investigated a flexible and skin-attachable SERS sensor that could detect bacterial pathogens without culture processes, as shown in Figure 7. Researchers fabricated a polymer NP array on a PET substrate by treating plasma and creating a plasmonic NP array by silver/gold deposition, as shown in Figure 7a. Each plasmonic NP had a height of 210 nm and a diameter of 75 nm. In this study, plasmonic NPs on a flexible film and gold nanoparticle tags that could be attached to Salmonella were simultaneously applied to perform enhanced Raman spectroscopic measurements of Salmonella on the skin. The co-localization of gold nanoparticle tags and a plasmonic NP array offered a higher plasmonic enhancement efficiency and SERS sensitivity for Salmonella detection. This SERS sensor could provide measurements in the range of 0–10^6^ CFU/mL, as shown in Figure 7b. Kang et al. [90] developed a plasmonic NP-fabricated SERS sensor for highly sensitive pH determination. In this study, plasmonic NPs were fabricated by stacking gold (Au)–SiO_2_ layers on a PET film using a soft lithographic technique. Then, the SiO_2_ layers in the plasmonic NPs were partially etched using an oxide etchant solution to form a dielectric nanogap between the Au layers. For pH determination, the researchers used 4-mercaptopyridine as the pH marker. Using Raman spectroscopy, which was significantly enhanced owing to the plasmonic NP array, they detected a small change in the molecular structure of 4-mercaptopyridine by varying the environmental pH. The researchers also employed multivariate regression as an analytical tool for the Raman-shift data to enhance the accuracy of SERS-based pH sensing. The results indicated that the developed SERS-based pH sensor enhanced by the plasmonic NPs could be used to analyze various liquid samples, such as beverages. He et al. [47] investigated flexible microfluidic nanoplasmonic sensors using plasmonic NPs to detect the biochemical components in sweat. After attachment to the skin, this flexible SERS biosensor could obtain biomedical information such as pH, lactate, and urea from sweat. A nanostructure for enhancing the accuracy of biochemical measurements performed using Raman spectroscopy was fabricated by sputtering a silver cap on a well-aligned silicone NP array. Using microfluidic techniques, researchers designed and established a skin-attachable fluidic biosensor to collect samples (sweat) from various areas in a measurement chamber, and plasmonic NP array substrates were fabricated in the chamber. Urea (1005 cm^−1^) and lactate (853 cm^−1^) were measured by label-free Raman spectroscopy enhanced by plasmonic NPs, and the pH was detected using 4-mercaptopyridine as a marker.

Additionally, several label-free cell-based assays using SERS have been developed using plasmonic NP-integrated cell culture substrates. Zhang et al. [91] developed plasmon-enhanced Raman spectroscopy to detect prostate-specific membrane antigens (PSMAs) in the plasma membranes of cells with high sensitivity. To enhance the sensitivity by field localization, the researchers used a plasmonic NP array coated with silver nanoparticles instead of a conventional metal-coated substrate. The plasmonic NPs used in this study were fabricated as follows: The self-assembled polystyrene beads were placed on a silicon wafer. After applying RIE to the wafer, Ag was deposited. After deposition, an NP array was formed using hydrofluoric acid and H_2_O_2_. A plasmonic NP array coated with silver nanoparticles was produced by applying ink containing silver nanoparticles. The plasma membrane of the cultured cells penetrated between the NPs; therefore, minute changes in the Raman signal from the molecular interactions in the plasma membrane could be acquired by plasmonic enhancements. The main result of this research was that SERS using plasmonic NPs coated with AgNPs could detect a difference in Raman signals from the plasma membrane in cells with and without PSMA, a biomarker of cancer. Additionally, the results of the cell viability assays confirmed that the plasmonic NP-integrated substrate was suitable for long-term (24 h) cell-culture experiments. Nam et al. [92] employed a plasmonic NP array to develop SERS for precisely detecting drug reactions in cells. In a previous study wherein plasmonic NPs were used for pH measurements [90], the SiO_2_ layers in the plasmonic NPs were partially etched using an oxide etchant solution to form dielectric nanogaps between the gold layers of each pillar. Additionally, SERS measurements enhanced by these nanogaps were employed to analyze the characteristic changes in the cell membrane and the responses to specific drugs with highly improved sensitivity. The researchers combined data from highly sensitive SERS measurements using plasmonic NPs with machine-learning algorithms to establish a platform that could identify the most suitable drug among multiple drugs.

### 4.3. High-Resolution Optical Imaging Using Plasmonic NPs

Research on the high-resolution optical imaging of microscale biomolecules in cells using dielectric and transparent NPs has been actively conducted since the early 2010s. In a representative study, Xie et al. [93] fabricated a cell substrate consisting of fused quartz NPs to form localized fluorescence excitations by optical illumination under the substrate [92]. The transparent NPs in contact with the cell membranes acted as optical waveguides that excited the fluorescence of small biomolecules in the cell membrane at a highly improved resolution. Combined with a conventional fluorescence microscope, the NP array-fabricated substrate provided high-resolution imaging of the intracellular microscale biomolecules (GFP–synaptobrevin) in cells cultured on the NPs. To improve the resolution of fluorescence imaging using transparent NPs, Roy et al. [94] utilized a silicone oil immersion objective lens and a three-dimensional single-molecule localization microscope for imaging a transparent NP-fabricated cell-culture substrate instead of a conventional optical microscope. Through this integration, three- and two-dimensional high-resolution biomolecular imaging for the transmembrane-labeled cells was obtained.

**Figure 8:**
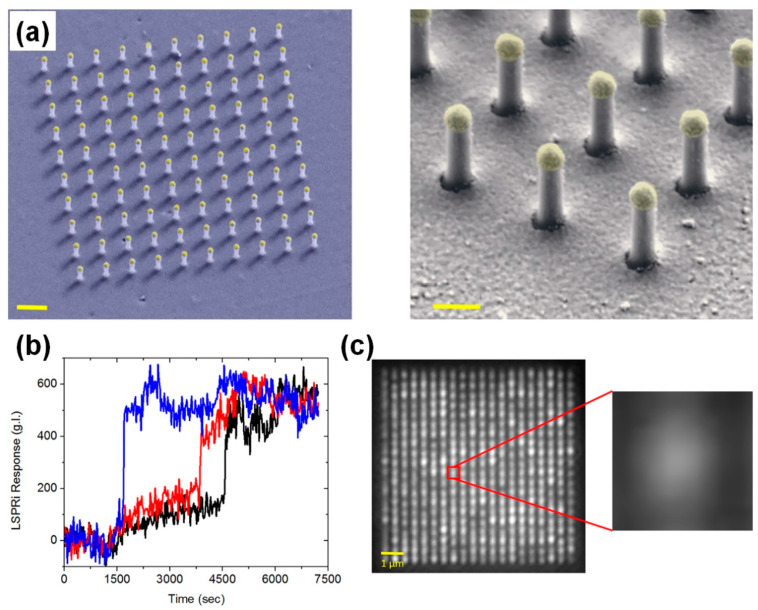
Application of plasmonic nanopillars to high-performance LSPR imaging for detection of a single exosome. (**a**) False-colored SEM images of the plasmonic nanopillars with a gold cap of 80 nm. (**b**) Spatially averaged intensities of LSPR images from three nanopillars when exosomes with a concentration of 1 × 10^5^/mL were inserted at 1200 seconds. (**c**) LSPR image of 20 × 20 plasmonic sensing arrays and a single sensing array. Reprinted with permission from Ref. [44]. Copyright 2018, Public Library of Science (PLoS).

Although it is not as effective as using plasmonic NPs for LSPR and SERS sensors, a few research groups have suggested plasmon-enhanced optical imaging using a plasmonic NP array with metal capping. Raghu et al. [44] applied plasmonic NPs to high-performance LSPR imaging for detecting a single exosome, as described in Figure 8. The single plasmonic NP had a diameter of 90 nm and a height of 490 nm, which included the height of a gold cap, which was 80 nm. Each plasmonic NP in the array had a period of 500–600 nm, and arrays of plasmonic NPs placed at 10 × 10 or 20 × 20 were fabricated with an interval of 25 µm. The diameter of the array-fabricated substrate was 25.4 mm. In combination with an SPR imaging system, the optical intensity could be measured at a specific illumination wavelength for each NP array, and LSPR images or spectra of the entire substrate could be acquired. This LSPR imaging system improved by plasmonic NPs could be used to acquire LSPR images and changes in the plasmonic signals of each NP array when exosomes with a concentration of 1 × 10^5^/mL entered the substrate as a sample. Additionally, this system provided a three-order sensitivity enhancement compared with that provided by the previously proposed real-time multiplexed SPR imaging system without NPs.

**Figure 9:**
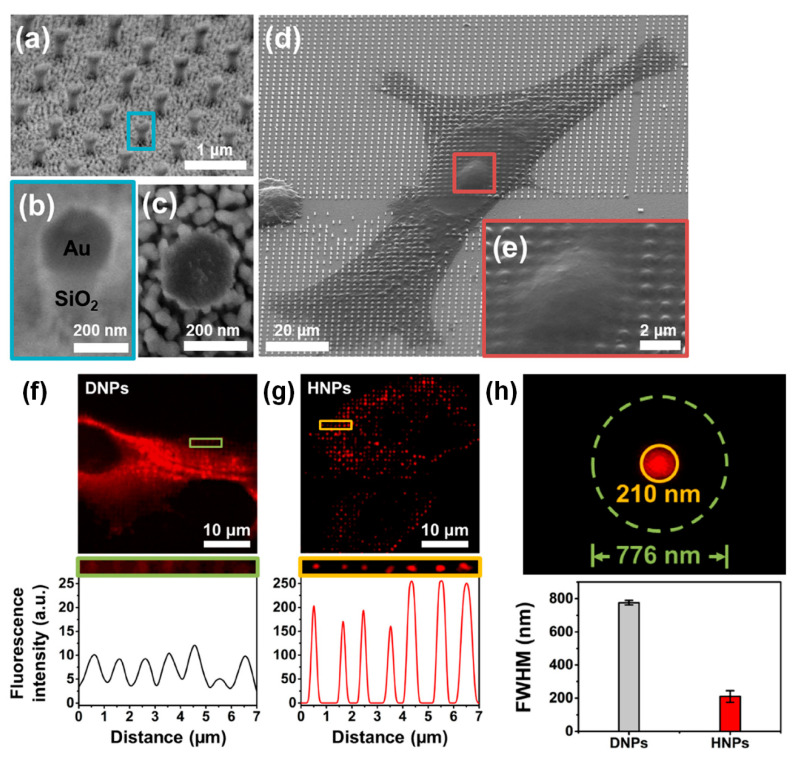
Development of high-resolution fluorescence imaging of cells using a plasmonic NP array. (**a**–**e**) SEM images of silica NPs with gold capping to obtain plasmonic resolution-enhanced fluorescence images. (**a**) Array of plasmonic NPs. (**b**) High magnification SEM image of a single plasmonic NP. (**c**) Top-view image of the single plasmonic NP. (**d**) A HeLa cell cultured on the plasmonic NP array. (**e**) Cell membrane deformation by plasmonic NPs. (**f**–**h**) Lateral-resolution enhancement of fluorescence imaging by the plasmonic NPs. (**f**) Fluorescence image of a HeLa cell cultured on a dielectric (silica) NP (DNP) array without gold capping and its intensity profile. (**g**) Fluorescence image of the HeLa cell on the plasmonic NPs with gold capping (hybrid NPs, HNPs) and its intensity profile. (**h**) Lateral full width at half maximum (FWHM) calculated from intensity profiles of fluorescence images using localized fluorescence excitations by DNPs and HNPs. As a result, the HNPs have offered improved lateral resolution in plasmon-enhanced fluorescence cell imaging. Reprinted with permission from Ref. [43]. Copyright 2020, De Gruyter.

Kim et al. [43] applied plasmonic enhancement to high-resolution fluorescence imaging using NPs to improve both the lateral and axial resolutions, as shown in Figure 9. To achieve plasmonic enhancement, a gold cap was deposited on silica (SiO_2_) NPs with a height of 700 nm, a diameter of 200 nm, and a period of 1 μm, as shown in Figure 9a–c. At an illumination wavelength of 633 nm, this plasmonic NP array had a localized field enhancement factor (|E|^2^ − 3000) approximately 40 times higher than that of silica NPs without the gold cap (|E|^2^ − 70). Additionally, the size of the plasmonic localized field (hot spot) produced by the plasmonic NPs was smaller than that of the light scattering field produced by the silica NPs without the gold cap. Based on this characteristic, fluorescence imaging enhanced by plasmonic NPs offered higher lateral and axial resolutions for microscale biomolecular imaging in cells. In conventional fluorescence imaging, this plasmonic NP array-fabricated substrate provided a plasmonic enhancement factor with a fluorescence intensity of 25. It was confirmed that the lateral full width at half maximum (FWHM) in the enhanced fluorescence imaging using plasmonic NPs was 210 nm, which was a significant improvement over the lateral FWHM provided by silica dielectric NPs (556 nm). In the application of the plasmonic NP-fabricated substrate to multiphoton imaging, the enhancement factor of the fluorescence intensity was 34, the lateral FWHM was 189 nm, and the axial resolution was 110 nm. This corresponded to super-resolution imaging, which broke the diffraction limit of conventional optical imaging.

## 5. Conclusions

**Figure 10:**
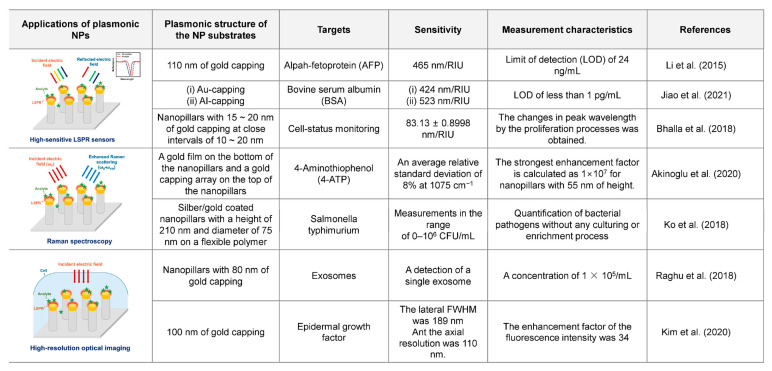
Overview of bio-applications based on plasmonic NPs: highly sensitive LSPR sensors, Raman spectroscopy, and high-resolution optical imaging [43,44,57,67,68,86,89].

This review focuses on research on employing plasmonic NPs for optical and biological applications in high-sensitivity LSPR sensors, Raman spectroscopy, and high-resolution imaging, as described in an overview diagram (Figure 10). Many researchers have used a basic structure with metal capping in dielectric NPs or various modified NPs to improve the sensitivity of biological sensing. For example, geometrical optimization was performed, wherein (1) the metal nanoislands were coated on the side of the NPs, (2) the height of the NP or the gap between the gold nanocappings was adjusted, and (3) artificial intelligence-based optimization was utilized to develop plasmonic NPs designed for enhanced sensitivity. Additionally, the physical flexibility of the materials can potentially be exploited when developing skin-attachable sensors.

These highly sensitive modified plasmonic NPs can efficiently measure biological molecules, such as intracellular markers. The NP structures can be adjusted by structural transformations in various directions, resulting in physical and geometrical progress toward improving the sensitivity performance. Introducing deep-learning technology will pave the way for new optimized methods for manufacturing NPs in various shapes with improved sensitivities compared to those of existing structures. Optimized plasmonic NPs can be employed in manipulating and investigating biological sensing, with a focus on diverse molecular events within or near the cell membrane. Specifically, membrane curvatures can be transformed locally into nanoscale regions created by the surface topography at the tips of NPs with high aspect ratios. They can be used as probes to easily analyze phenomena that occur near the cell membrane, such as endocytosis and the distribution of the cytoskeleton. Applying LSPR-based NP sensing is expected to be useful in applications requiring real-time measurement and the analysis of bio-targets with highly-improved sensitivity.

## Data Availability

Data sharing is not applicable.
